# Construction Notes

**Published:** 1894-01-20

**Authors:** 


### CONSTRUCTION NOTES.

**Tooting Fever Hospital.**

The architect for this new fever hospital was Mr. Aldwinckle. The ground being bought by the Metropolitan Asylums Board consists of 32 acres. This site is divided by Grove Road into two unequal portions, on one of which, comprising about ten acres, the temporary hospital is now being erected. The larger portion is reserved for the permanent structure to be forthwith proceeded with. The building provides accommodation for 390 patients in the ordinary pavilion, besides sixteen beds in two isolation blocks. Room will be found for eighty nurses, maid servants, and fifteen male servants, as well as for officers. When we consider the stores, the kitchens, the wash-houses, and laundries, the heating, the engines, the disinfecting arrangements, the supply of gas, water, and steam, the complete system of drainage, with manholes, ventilating traps, and flushing tanks, for the whole of this ten acres of land and buildings, we then begin to appreciate the greatness of this undertaking. The mode of construction is practically the same for all the building. Each pavilion consists of a large ward, 26 ft. by 144 ft., for twenty-four patients, a small ward with one bed only, a nurses' duty-room containing a small range and a well-ventilated meat safe for provisions, bath-room, linen store-room, and two water-closets for patients. In each large ward there are four open ventilation grates placed in pairs back to back, each connected with the open air by a nine-inch drain-pipe; under every bed is a direct opening to the outer air. The hospital is intended for the treatment of only one disease—scarlet fever. A series of isolation wards in which doubtful cases may wait for maturity is also incorporated in the scheme. A large recreation-room will be provided for the nurses.

